# Handgrip and quadriceps muscle endurance testing in young adults

**DOI:** 10.1186/2193-1801-2-451

**Published:** 2013-09-11

**Authors:** Ciara White, Kimberley Dixon, Dinesh Samuel, Maria Stokes

**Affiliations:** Faculty of Health Sciences, University of Southampton, Highfield, Southampton SO17 1BJ United Kingdom

**Keywords:** Grip endurance, Quadriceps, Maximal intermittent contraction, Isometric, Muscle function

## Abstract

**Background:**

Grip strength is widely used for estimating whole body strength but there is a lack of information relating to grip endurance. Comparison between endurance of different muscle groups has received little attention. The main aim of the present study was to determine the endurance characteristics of hand grip and quadriceps muscles in healthy young adults and then to examine the association between fatigability of the two muscle groups.

**Methods:**

Twenty one healthy participants (8 males and 13 females) aged 18–35 years were studied. A maximal intermittent endurance test, consisting of 12 isometric contractions held for 3 seconds separated by 5 second rest periods, was utilised to measure muscle endurance. A Biodex isokinetic dynamometer and Jamar dynamometer were used to assess quadriceps and hand grip respectively. The mean of first (M_1_) and last (M_2_) three repetitions was calculated. Fatigue index values were calculated for both muscle groups by the 1st peak torque (PT) minus the last (12th) PT, divided by the 1st PT multiplied by 100.

**Results:**

Quadriceps torque (M_1_:197.3 ± 65.2 Nm; M_2_:163.1 ± 47.6 Nm) and grip strength (M_1_:33.6 ± 9.9 Kg; M_2_:25.2 ± 8.1 Kg) both declined significantly during the 12 repetitions (p < 0.05). Hand grip showed a significantly higher mean fatigue index of 30% compared to 18% in the quadriceps (p < 0.05).

**Conclusions:**

Quadriceps showed better fatigability than hand grip. The findings therefore indicate caution against using grip endurance as a surrogate measure of quadriceps endurance. Further research is warranted to confirm observed differences between genders and to study endurance in different age groups.

## Introduction

Grip strength is widely used due to the portability and practicality of grip dynamometry (Bohannon 2002). Grip strength has been shown to be a predictor of general body strength, postoperative complications, mortality and functional decline (Bohannon 2001; Bohannon 2009; Boissey et al. 1999; Massey-Westrop et al. 2004).

Many activities of daily living (ADL) require a sustained effort exerted over a period of time. Therefore, muscle endurance is an important aspect of physical performance and needs to be considered when assessing musculoskeletal function. Recent studies have examined the association between impairments of muscle endurance and underlying pathology relating to morbidity (Hulsmann et al. 2004; Januadis-Ferreira et al. 2006; Coronell et al. 2004). Particular emphasis was placed on the association between respiratory problems, such as chronic obstructive pulmonary disease, and quadriceps endurance (Januadis-Ferreira et al. 2006; Coronell et al. 2004; Van’t Hull et al. 2004; Allaire et al. 2004).

Suitable measurement protocols need to be established to quantify endurance in clinical and epidemiological settings. Differing methodologies and lack of consistency in the use of outcome measures for quantifying muscle endurance, makes comparisons between studies difficult. For example, studies on muscle endurance have used various equipment and protocols, including maximal intermittent contractions (Taylor et al. 2000) and sustained submaximal contractions (Januadis-Ferreira et al. 2006; Van’t Hull et al. 2004; Allaire et al. 2004; Swallow et al. 2007). Intermittent contractions cause slower loss of force than sustained contractions (i.e. less fatiguable), so the results produced by the two types of protocol cannot be compared (Taylor et al. 2000).

Previous research on grip endurance utilised a sustained sub-maximal measurement protocol, representing 50% of the participant’s maximal voluntary contraction (MVC) (Desrosiers et al. 1997; Reuter et al. 2011). A grip dynamometer was utilised but no visual feedback was provided to participants. The above methodology had limitations, as it required high levels of concentration and motivation on the part of the participant, and consistent verbal encouragement from the assessor was needed as some participants could not effectively maintain the exact target (Desrosiers et al. 1997). Maximal intermittent tests involving a 10- repetition maximal contraction was used previously to quantify grip endurance (Nicolay and Walker 2005). Endurance was quantified by comparing the mean value of the first 3 repetitions to the mean value of the last 3 repetitions, and most participants showed a significant decline over the 10 repetitions (Nicolay and Walker 2005).

An investigation using the isometric maximal intermittent endurance test of four muscle groups; finger flexors, thumb abductors, dorsiflexors and plantarflexors, reported significant differences in endurance among the four muscle groups, with lower extremity muscles having a greater endurance capacity than those of the upper extremity (Bemben et al. 1996). There is some evidence on endurance of different muscle groups but given that grip strength is used as a surrogate for quadriceps strength, comparative data on the endurance of the two muscle groups is lacking.

Therefore, the aim of the present study was firstly, to quantify the muscle endurance characteristics of grip and quadriceps muscles and secondly, to examine the association between quadriceps and grip endurance in young adults.

## Methods

An exploratory study utilising an experimental design was conducted to investigate muscle endurance in healthy young adults. Ethical approval was obtained from the Faculty of Health Sciences Ethics Committee at the University of Southampton. All participants gave written, informed consent.

### Participants

A convenience sample of 21 healthy participants (8 males and 13 females), aged 18–35 years was studied. Participants who were recreationally active and who had full pain free range of motion at the knee and wrist were included. Those with musculoskeletal or neurological conditions or injury to the lower or upper limbs in the previous six months and those under current medical treatment were excluded. Participants were recruited from the University of Southampton through poster advertisements.

### Testing protocol

Participants underwent testing of the dominant upper and lower limb on one occasion only. Limb dominance was established by asking which leg they would kick a football with and which hand they would predominantly write with. Height and weight were measured using appropriate scales. All participants performed a sub-maximal warm up of both quadriceps and grip muscles, consisting of 8–10 repetitions on a Biodex dynamometer and 3–5 repetitions on the Jamar dynamometer respectively. The order of testing was randomised in order to minimise bias in the fatigue effects, and half the group performed the quadriceps test first, followed by the grip test and vice versa. The second handle position was utilised for grip endurance measurements. The test procedures were standardised and data collection was carried out by the same tester. All data collection was carried out in the Biomechanics laboratory in the Faculty of Health Sciences, University of Southampton.

### Quadriceps endurance

The Biodex system II dynamometer (Biodex medical, New York, N.Y., USA) was used to assess quadriceps endurance. A calibration check was performed in accordance with the manufacturer’s specification. Participants were positioned with their upper body strapped firmly against the back rest of the chair, with the hips and knees at 90° and straps tightened across their chest, hips and thighs. Quadriceps endurance test consisted of 12 maximal isometric knee extensions of the dominant leg. Contractions were held for 3 seconds, with a 5 second rest period between each repetition. A traffic light system on the computer screen and standardised verbal commands from the researcher ensured participants were contracting at the appropriate times. Endurance was assessed using the percentage fatigue index value, calculated by the 1st peak torque (PT) minus the last (12th) PT, divided by the 1st PT multiplied by 100. In addition, the percentage change of torque (mean of last three repetitions/mean of first three repetitions) was computed (Nicolay and Walker 2005).

### Grip endurance

Grip endurance was measured using an electronic JAMAR dynamometer (Biometrics, UK). Participants were seated with their shoulders at 0° abduction and neutral rotation, elbows at 90° flexion and their forearms in the neutral position (ASHT 1992). The protocol of 12 contractions was also used to test grip endurance. Data obtained from the Jamar dynamometer were analysed using MATLAB. The peak strength values were computed and endurance was expressed as the percentage fatigue index, calculated in the same way as for the quadriceps data. In addition, the percentage change of force was calculated as described in the previous section. Visual feedback was provided to participants and verbal encouragement was standardised throughout both testing protocols.

### Data analysis

Statistical analysis was carried out using SPSS version 19.0. Data were tested for normality using the Shapiro-Wilks test, and found to be normally distributed. A paired t-test examined the differences in mean values of the first three (M_1_) and last three (M_2_) repetitions. Differences between males and females were assessed using an independent samples t-test. Pearson’s correlation coefficient and linear regression were used to assess the relationship between endurance of the two muscle groups. The percentage fatigue index values were used in the analysis, and a paired t-test examined any significant difference between muscle groups.

## Results

### Participant characteristics

The study included a total of 21 participants, 8 males and 13 females with a mean age of 23.5 (SD 1.4) years. The means, standard deviations (SD) and ranges for height (cm), weight (kg) and BMI are presented in Table 1.

**Table 1:** **Participant characteristics**

	Males		Females		All participants	
	***n = 8***		***n = 13***		***n = 21***	
	Mean (±SD)	Range	Mean (±SD)	Range	Mean (SD)	p-value
**Age**	24.1 (1.7)	22- 27	23.1 (1.0)	21-24	23.5 (1.4)	.094
**Height**	180.5 (8.5)	166-191	166.9 (9.9)	153-186	172.1 (11.4)	.005*
**Weight**	79.5 (9.1)	67-98	64.2 (9.3)	53-84	70.0 (11.8)	.002*
**BMI**	24.3 (1.4)	22.3-26.9	22.9 (1.5)	20.7-25.1	23.5 (1.6)	.04*

(*indicates a significant difference between males and females, where p < 0.05).

The quadriceps and grip endurance values including data on strength normalised to body weight for the first and last three repetitions are reported in Table 2.

**Table 2:** **Summary of grip and quadriceps endurance values during the first three and last three repetitions**

			Grip			Quadriceps		
	Mean of first three repetitions (Kg)	Mean of last three repetitions (Kg)	Mean of first three repetitions normalised to body weight	Mean of last three repetitions normalised to body weight	Mean of first three repetitions (Nm)	Mean of last three repetitions (Nm)	Mean of first three repetitions normalised to body weight (Nm/Kg)	Mean of last three repetitions normalised to body weight (Nm/Kg)
	Mean (±SD)	Mean (±SD)	Mean (±SD)	Mean (±SD)	Mean (±SD)	Mean (±SD)	Mean (±SD)	Mean (±SD)
**All**	33.6 (±9.9)	25.2 (±8.1)	0.3 (±0.2)	0.2 (±0.2)	197.3 (±65.2)	163.1 (±47.6)	2.8 (±0.6)	2.3 (±0.5)
**Males**	42.4 (±6.2)	32.7 (±5.2)	0.5 (±0.2)	0.4 (±0.2)	269.4 (±33.9)	209.3 (±33.9)	3.4 (±0.3)	2.6 (±0.3)
**Females**	25.9 (±4.2)	18.7 (±2.0)	0.2 (±0.2)	0.2 (±0.2)	152.9 (±28.5)	134.7 (±28.5)	2.4 (±0.4)	2.1 (0.4)

### Maximal intermittent quadriceps endurance (MIQE)

**Figure 1:**
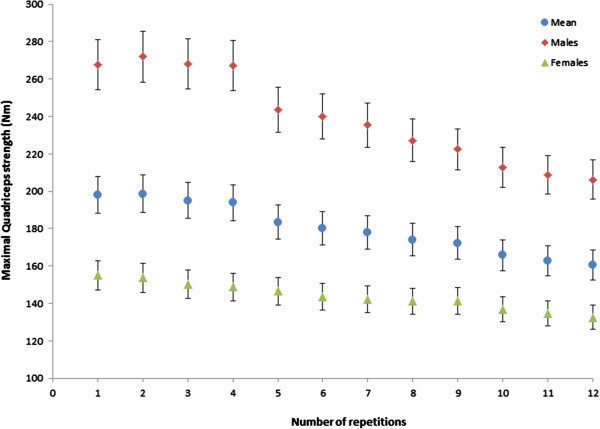
**Mean decline in torque over 12 repetitions during the maximum intermittent quadriceps test.**

The peak torque values were obtained from each of the 12 maximal repetitions. The knee extensor torque declined during the maximal intermittent test (Figure 1) and the mean value of the last three repetitions 163.1 Nm (SD 47.6) was significantly lower (p < 0.05) than the mean of the first three repetitions 197.3 Nm (SD 65.2) (Table 2). The mean fatigue index was 18.1% (SD 9.7) and males (22.8%; SD 9.6) had higher values (p < 0.05) compared to females (15.2%; SD 8.9), indicating greater fatigue (Table 3).

**Table 3:** **Grip and quadriceps endurance (% Change in force = mean of last three repetitions/mean of first three repetitions) and fatigue index**

	% Change in grip strength	% Change in quadriceps torque	Grip fatigue index^+^	Quadriceps fatigue index*
	(Kg)	(Nm)		
	Mean (±SD)	Mean (±SD)		
**All**	75 (±7.2)	84.1 (±9.5)	29.8 (±10.9)	18.1 (±9.7)
**Males**	77.1 (±5.3**)**	77.9 (±9.6)	28.1 (±8.3)	22.8 (±9.6)
**Females**	73.2 (±8.5)	87.9 (±7.4)	31.2 (±13.1)	15.2 (±8.9)

* Significantly different between males and females (p < 0.05).

^+^ Grip had a significantly higher mean fatigue index compared to quadriceps (p < 0.05).

### Maximal intermittent grip endurance (MIGE)

**Figure 2:**
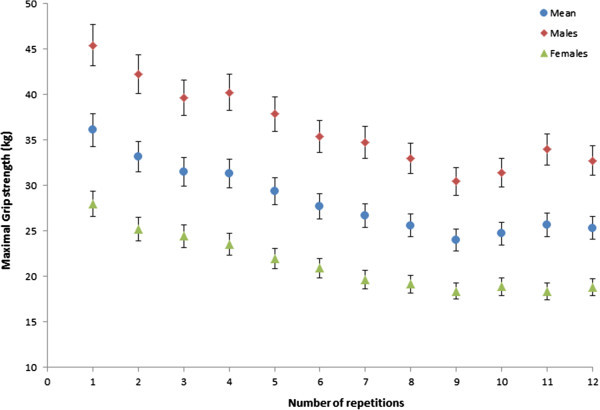
**Mean decline in force over the 12 repetitions during the maximum intermittent grip endurance test.**

**Figure 3:**
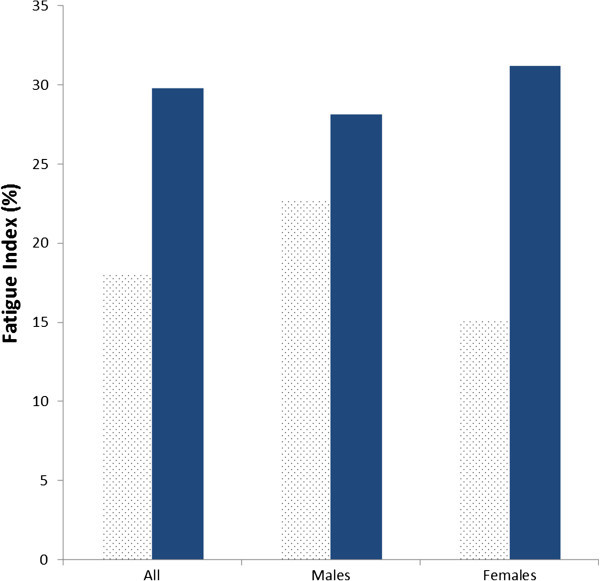
**Percentage fatigue index for handgrip (Dots) and quadriceps (Solid).**

A decline in grip strength was noted during the maximal intermittent test (Figure 2) and the mean value of the last three repetitions 25.2 kg (SD 8.1) was significantly lower (p < 0.05) than the mean of the first three repetitions 33.6 kg (SD 9.9) (Table 2). The mean fatigue index was 29.8% (SD 10.9) and there was no significant difference between females (31.2%; SD 13.1) and males (28.1%; SD 8.3) (Figure 3).

### Relationship between grip and quadriceps endurance

Pearson’s correlation indicated a low (r = -0.090), negative correlation between the percentage fatigue index values of the two muscle groups. A linear regression between the fatigue index values with quadriceps as the dependent variable provided an R^2^ value of 0.068 (p = 0.75). When BMI was included as a dependent variable an R^2^ value of 0.184 (p = 0.51) was obtained.

## Discussion

The present study provides comparative data on the fatiguing characteristics of grip and quadriceps muscles in a sample of healthy young adults. The results highlight that the quadriceps muscle group may have a greater ability to maintain endurance in comparison to hand grip. In addition, males appeared to show greater fatigue than the females for quadriceps but the numbers are too small to draw a definitive conclusion on the effect of gender. Previous research has indicated that men were more fatigable than women during both intermittent and sustained contractions (Hunter et al. 2004). Women had a significantly longer time to task failure (1408 ± 1133 s vs 513 ± 194 s, p < 0.05) compared to men (Hunter et al. 2004).

The differences in endurance between the two muscle groups suggest that caution should be taken when considering using a grip endurance measurement as a surrogate for quadriceps endurance and vice versa. This is in line with previous literature on the association of maximal isometric strength measurements between quadriceps and handgrip (Samuel and Rowe 2011; Bohannon 2008; Samuel et al. 2012). The findings of present study concur with previous research on different muscles, which reported that the muscles of the lower extremity had greater endurance capacity than the muscles of the upper extremity (Bemben et al. 1996). Our findings were largely unaltered when muscle strength was normalised to body mass with the exception of grip endurance in women where the mean of first and last three repetitions was similar. During the present grip endurance task, a fatigue index of 30% was reported, while a considerably lower value of 18% was noted for the quadriceps. This indicates that the grip may fatigue at a higher rate than the quadriceps and may be attributed to differences in the physiological make-up of the two muscle groups. Maximal contractions were used in the present study to assess their utility for clinical testing and depended primarily on anaerobic metabolism and type II fast twitch muscle fibres (Lexell 1995). The ability to maintain adequate levels of endurance depend on physiological factors including fibre type composition, muscle blood flow and maximum force of the muscle group tested (Bemben 1998; Enoka and Duchateau 2008; Hunter et al. 2004). The fast twitch fibres have greater energy demands but lower capability for oxidative phosphorylation compared with the slow twitch fibres. Hence the proportion of different fibre types will affect the fatigue characteristics of a muscle (Wust et al. 2008). The fibre type composition of quadriceps is 50:50 (type I: type II), and with larger motor units and contractile elements, it has a greater potential for muscle performance (Wynsberghe et al. 1995). This is in comparison to handgrip which utilises a much smaller collection of muscles, with smaller motor units available for contraction and higher proportion of type 1 fibres (Wynsberghe et al. 1995). Therefore, they are more likely to fatigue at a faster pace. Furthermore, maximal contractions were used in the present study and the high level of contraction required should be taken into consideration. For instance, if 20% of maximal voluntary contraction had been used, this would have favoured slow twitch muscles, so hand grip may have shown greater endurance than quadriceps. Endurance protocols therefore need to consider the makeup of muscles and it may not be valid to use the same protocol for muscles that have different contraction characteristics.

In addition, factors possibly influencing the results include gender, motivation, extent of verbal encouragement and feedback provided to the participants (Capodaglio et al. 1997). As described earlier, gender was analysed within the two endurance tests, and males showed a greater decline in isometric strength when compared to females in the maximal intermittent quadriceps test. On the contrary, the grip fatigue index values were not significantly different between males and females. The fatigue indices showed higher variability compared to muscle strength and this might explain the lack of relationship between quadriceps and grip endurance. These observations need to be repeated in larger numbers, to provide conclusive evidence of a difference in endurance between genders, ages and muscle groups.

Due to the fact that a maximal effort was required during the endurance tests, participant motivation was vital. However, maximal values were not confirmed by twitch interpolation. Hence, verbal instructions were standardised to encourage maximal force production (McNair et al. 1996). Effort may have been sub-maximal for grip as the muscle fatigued, as an increase in force was observed between repetitions 10–11 (Figure 2). However, this was not the case for quadriceps torque, which declined throughout the protocol. A few participants reported mild discomfort during the grip endurance test. Similar limitations with equipment comfort were reported in previous studies (Nicolay and Walker 2005).

It would be beneficial to add a grip endurance measurement using protocols that can be replicated in a clinical environment. The maximal intermittent protocol was used rather than a sub-maximal endurance test, as maximal contractions would be simpler to perform in clinical situations. In addition, it is difficult to perform a sub-maximal test without visual feedback of a target and not all systems provide this feedback. Maximal tests might be more relevant to older people who may be working at a higher proportion of their available strength during everyday activities (Samuel et al. 2011). However, as mentioned earlier endurance protocols may need to consider the physiological makeup of a muscle and the same protocol may not be appropriate to test different muscles when contraction characteristics differ.

## Conclusions

The present findings provide preliminary data on the fatigue index, indicating the endurance capacity of two important muscle groups, handgrip and quadriceps, in healthy young adults. Due to the increasing proportion of older adults in the population, it would be beneficial to test the endurance protocols in this group. Furthermore, larger studies are needed to provide reference data for groups of healthy males and females across the age spectrum. A measure of muscle endurance along with maximal strength may provide a more accurate picture of muscle function and act as an objective marker for rehabilitation.
